# Alkali-Activated Binder of Municipal Solid Waste Incineration Bottom Ash at Lower pH Levels

**DOI:** 10.3390/ma18051076

**Published:** 2025-02-27

**Authors:** Gintautas Tamošaitis, Danutė Vaičiukynienė

**Affiliations:** Building Materials and Structures Research Centre, Faculty of Civil Engineering and Architecture, Kaunas University of Technology, Studentu st. 48, Kaunas LT-51367, Lithuania; danute.vaiciukyniene@ktu.lt

**Keywords:** MSWI bottom ash, silica gel waste (SW), alkali activation

## Abstract

This paper focuses on the alkaline activation of municipal waste incineration (MSWI) bottom ash to create a dense, non-porous composite structure. Normally, high pH solutions are used to activate MSWI bottom ash, but this has the side effect of creating residual effects in the bottom ash. Due to the uniqueness of the incineration process, the bottom ash retains metallic aluminum, which reacts with the alkali to produce hydrogen gas, which forms a porous structure in the sample during the hardening of the composite. This study demonstrates a method of eliminating this effect by lowering the pH of the alkaline activator below 12.5. An alkali-activated binder was prepared from ground MSWI bottom ash as a precursor and a triple alkali activator: NaOH solution, soluble glass (SG) and silica gel waste (SW). The highest compressive strengths of about 20 MPa were achieved for alkali-activated MSWI bottom ash with triple alkali activators such as sodium hydroxide, soluble glass and silica gel waste.

## 1. Introduction

Bottom ash is the solid residue that results from the combustion of municipal or industrial waste in incinerators to produce electricity and heat for the urban heating system. In this process, about 25% of the original mass of the waste remains as bottom ash. There are currently three main incineration plants in Lithuania which generate this ash. The amount of MSWI bottom ash is steadily increasing year by year, creating a problem for the recovery of this ash.

According to Chen et al. [1], bottom ash from municipal solid waste incineration (MSWI) is possible to use as supplementary cementitious material (SCM) and alumina–silica precursor for alkali-activated binders. Most investigations have reported that geopolymers containing MSWI ash exhibit low strength due to low reactivity (insufficient amount of reactive silicon and aluminum compounds) and the presence of metallic aluminum. During the alkaline activation process, aluminum reacts with sodium hydroxide (at high pH solution) to form hydrogen, which adversely affects mechanical properties [2]. In our previous study [3], MSWI ash was used as an aluminosilicate precursor and porous-creating agent as well, wherein 15% of phosphogypsum acted as a binding accelerator of the porous alkali-activated system. In this case, the samples had a compressive strength of 1.8 MPa, the density reached 900 kg/m^3^ and the thermal conductivity was 0.23 W/(m × K).

So, a porous structure and limited amount of geopolymerization products negatively affected the strength properties of geopolymers based on MSWI ash. The quality of MSWI bottom ash can be improved through chemical, mechanical and thermal treatment. The study conducted by Carvalho et al. [4] suggested using fast carbonation of the alkali-activated ash samples. The release of hydrogen caused the samples to expand and reduce their strength properties. This carbonization neutralized the metallic aluminum, which reacted with sodium hydroxide to produce hydrogen in the samples without carbonization. In another study by Han et al. [5], they investigated a geopolymer binder made from MSWI ash, coal fly ash and metakaolin, and found that the samples showed a similar increase in porosity. The addition of 40% MSWI ash in the blends resulted in an 80% reduction in compressive strength. With less than 15% MSWI ash, an increase in strength was observed at early hydration ages (up to 7 days), while a negligible effect was observed at longer ages (7–28 days). This can be explained by the formation of a lower amount of sodium and calcium aluminosilicate hydrate gel, with increases the porosity. A different composition of the precursor was developed by Lin et al. [6]. In this case, the precursor was based on MSWI ash, coal fly ash and ground granulated blast furnace slag blends. After 28 days of curing, these geopolymers had a compressive strength of more than 60 MPa. The resulting blends increase the reactivity of the precursors with MSWI ash due to the synergistic effect between the precursor ingredients. Diaz-Loya et al. [7] concluded that MSWI fly ash and coal fly ash, consisting of 80% and 20%, respectively, reached a compressive strength of 15.8 MPa after alkaline activation with NaOH and water glass. Coal fly ash has been used as an initial material for precursors rich in the active form of silica and aluminum compounds. According to the study of Huang et al. [8], MSWI ash and ground granulated blast furnace slag blends have a low amount of silicon compounds in the active form, resulting in compressive strengths of up to 15 MPa and low levels of geopolymerization products. The strength values were improved (50 MPa, after 28 days) when samples were formed with soluble glass at about 26 wt%. Soluble glass led to the formation of C-A-S-H and C-S-H gels in the alkali-activated binder system. In the study [9], the fly ash was obtained from a municipal waste incineration plant in Poland. The use of this fly ash and coal fly ash as the precursor of geopolymers does not provide materials with sufficient strength parameters (less than 0.6 MPa of compressive strengths).

The mechanical properties depend not only on the properties of the precursor but also on the type and number of alkaline activators. Morsy et al. [10] found that the best ratio of sodium silicate to sodium hydroxide for alkali-activated fly ash is 1.0. The highest mechanical properties were achieved with a compact microstructure of this composition. Liu et al. [11] investigated two types of MSWI ash: fly ash and bottom ash. The experimental results show that, when the precursor is dominated by MSWI fly ash, the samples reach their maximum strength (8.8 MPa) after alkali activation after 56 days at a lower alkali concentration (6% Na_2_O). In contrast, MSWI bottom ash requires a higher concentration of alkali; for example, 8% Na_2_O. This can be explained by the fact that, in alkaline-activated MSWI fly ash, the main hydration compound is the C-S-H gel, whereas in alkaline-activated MSWI bottom ash, the C-(A)-S-H gels and N-A-S-H gels predominate. The types of hydration products indicate that the activation of bottom ash by alkalis requires the use of a larger number of alkalis. Moreover, MSWI bottom ash contains chemical compounds in a nearly inert state: quartz and calcite, according to Maldonado-Alameda et al. [12]. For this reason, the compressive strength of alkali-activated MSWI bottom ash does not exceed 10 MPa. Thus, the compressive strength of alkali-activated samples made from MSWI bottom ash or/and MSWI fly ash can vary over a very wide range, from 0.3 MPa to 160 MPa, depending on the method of neutralization of the metallic aluminum. Kurda et al. [13] found that the best way to neutralize metallic aluminum is by electromagnetic MSWI ash separation.

In this study, it was proposed to use a pH value below 12.5 for the starting mixtures, which do not release hydrogen gas. This study proposes the use of ground MSWI bottom ash as a precursor for the preparation of an alkali-activated binder. Sodium hydroxide combined with sodium silicate and silica gel waste was used as alkali activators.

## 2. Characterization of Initial Materials

The ash from municipal solid waste incineration (MSWI) was used for this test as precursor. The municipal waste is incinerated at a temperature of 1200–1600 °C. The remaining bottom ash is a viable material that can be used or properly treated in alkaline-activated composites. The ash consists of incompletely burnt or completely unburnt materials, including mineral and metallic fractions. They are non-hazardous ashes in an inert state. After the incineration process, the bottom ash is stored in open areas where it is sorted by fraction and the majority of the metal fraction is separated for recycling or reuse. However, some metals (iron, copper, aluminum) cannot be completely separated from the ash by mechanical means. For this study, the bottom ash was taken from the storage site before the sorting process and only the larger metal pieces were removed from the ash. In the laboratory, the unsorted ash was dried at 100 °C and milled in two stages; first in a large-volume vibratory mill and then again in a small-volume ball mill. This gives the highest level of ash milling. The Blaine of specific surface area for ground MSWI ash was 352 m^2^/kg. XRF analysis of MSWI ash oxides shown in Table 1.

**Figure 1:**
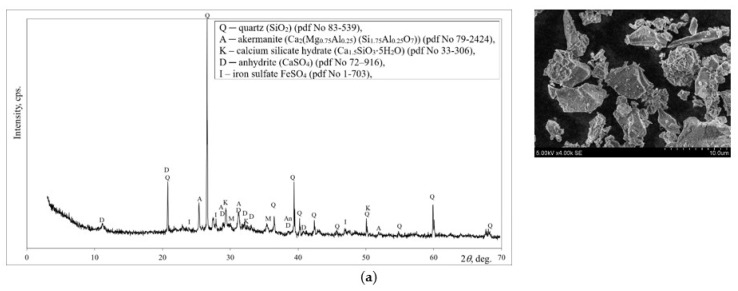

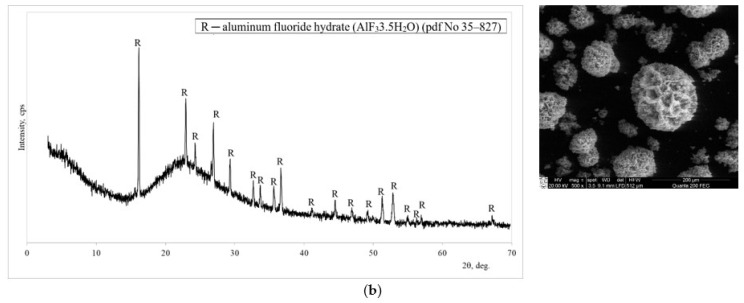
Mineral composition and microstructure of bottom ash (MSWI) (**a**) and silica gel waste (**b**).

The microstructural examination (SEM) showed that the particles of ground MSWI ash are irregularly shaped with sharp edges (Figure 1a). In the mineral composition of MSWI ash (XRD analysis), quartz and akermanite dominated, with a small amount of C-S-H, anhydrite and iron sulfate. Similarly, the mineral composition of MSWI bottom ash was determined by Bayuseno et al. [14].

Three different types of alkaline activators were used: sodium hydroxide, sodium silicate and silica gel waste. Sodium hydroxide combined with sodium silicate is the most popular alkali activator [11]. In this case, the alkali activator was made from the combination of sodium hydroxide, sodium silicate and silica gel waste. The first type of alkaline activator is NaOH as commercial reagent in granular form (99% purity, DeltaChem, Praha, Czech Republic). The second type of alkaline activator which was used is soluble glass sodium silicate hydrate (Silpur, Wrocław, Poland) with silicate modulus 3.0 and ρ = 1290 kg/m^3^. Finally, the third type of activator is silica gel waste. This by-product (silica gel waste) is produced by a fertilizer plant JSC Lifosa in Lithuania, generating about 15,000 tons per year. The silica gel waste is composed of a fine powder and it is dried at 100 °C before the alkali activator is prepared. The Blaine-specific surface area for silica gel waste was about 716 m^2^/kg. In terms of chemical composition (XRF analysis), this silica gel waste is dominated by SiO_2_, which accounts for about 77.59% by weight. Fluoride accounts for about 7.24% by weight and alumina for 4.12% by weight. The content of other oxides does not exceed 1% by weight. XRD analysis showed that aluminum fluoride hydrate (AlF_3_·3.5H_2_O) predominates as a crystalline compound (Figure 1b). Silica gel is amorphous and, in addition to this crystalline compound, the amorphous part can also be distinguished: an amorphous hallo peak was detected at 15–34 (2θ). SEM analysis shows that the particles are irregularly shaped and have a porous structure. These fertilizer production wastes are almost constant in elemental and mineral composition. Other studies [15,16] have also shown that the same crystalline compounds (AlF_3_·3H_2_O) and similar elemental composition (77.77 %wt) predominate.

## 3. Quantities of Initial Materials for the Preparation of Mixtures

Each mixture consisted of two parts: MSWI powder (precursor), which was dried and grounded, and an alkali activator. The activator consisted of a solution of soluble glass, sodium hydroxide and silica gel waste in varying amounts. The activator components had different water contents or were fully dried. The silica gel waste was fully dried and had no additional water content. The sodium hydroxide used was in dry granular form. In the preparation of the alkaline activator, the pH of the individual components was determined. The pH of solutions for soluble glass was 11.4, for sodium hydroxide it was 13.4 and for silica gel waste it was 2.5. Silica gel waste is acidic and opposite to the activation of alkaline mixtures. These pH levels were determined by dissolving the starting activator materials in water. Table 1 shows the stepwise changes in the pH of the activator. The pH of the sodium silicate solution was measured first. The pH of the sodium hydroxide granules dissolved in the sodium silicate solution was then measured. This activator solution was used in the control sample M0 to show the effect of a high pH level on the mixture. A third measurement of the pH of the activator solution was carried out by dissolving silica gel waste in a solution of sodium silicate and sodium hydroxide. It is this component of the activator that sufficiently lowers the pH of the solution below pH 12.5. The water-to-solid ratio (W/S) was in the rage of 0.2–0.4. Varying the proportion of components in the activator solution results in different mixtures. The compositions of the mixtures are given in Table 2.

The activator solutions prepared according to the prescribed proportions were mixed with MSWI powder to form a paste. The prepared mixtures were poured into plastic molds of 20 × 20 × 20 mm^3^ and were sealed in polythene to prevent the evaporation of water for 28 days. The first day, the materials were cured at 20 °C, the second day at 60 °C and finally the samples were stored in at 20 °C for 26 days. It is known that samples cured in an oven have higher strengths than those cured at ambient temperature [17]. A previous study [18] found that a curing temperature of 60 °C and a curing time of one day is optimal for achieving higher strengths. A paste made from alkaline activator and MSWI bottom ash was aged for one day at an ambient temperature. This ageing process was useful for the formation of zeolitic structures and accelerated the crystallization process [19,20]. During the ageing process, the seeds of zeolitic structures form inside the gels, which improves the repolymerization process.

## 4. Experimental Techniques

The X-ray diffraction analysis (XRD) was used to assess the mineral composition of the precursor and hydration products after alkali activation. It was carried out using a D8 Advance diffractometer (Bruker AXS, Karlsruhe, Germany) operating at the tube voltage of 40 kV and tube current of 40 mA. X-ray fluorescence (XRF) was conducted for elemental analysis. It was performed on an X-ray fluorescence spectrometer Bruker X-ray S8 Tiger WD (Karlsruhe, Germany). The specific surface area was measured using the Blaine method EN 196-6:2018 standard [21]. The microstructures were studied by using a high-resolution scanning electron microscope FEI Quanta 200 FEG with a Schottky field emission gun (FEG) (Hillsboro, OR, USA). The pH measurements of water suspensions were conducted with the pH meter EDGE, 230 V (Washington, DC, USA). Compressive strength was determined after 7 and 28 days of hydration. A hydraulic press according to EN 196-1 was used to determine the compressive strength.

## 5. Results and Discussion

For the reaction between aluminum and sodium hydroxide to take place, the pH of the solution must be strongly alkaline, typically above 12.5. At this high pH level, the hydroxide ions (OH^−^) are sufficiently concentrated to dissolve the thin protective layer of Al_2_O_3_ that normally protects the aluminum surface and allows the reaction to proceed [22]. Aluminum reacts with NaOH in the presence of water to form sodium aluminate (Na[Al(OH)_4_]) and to give off H_2_ gas, which makes the samples porous. This can be clearly seen in the M0 (control) mixture samples (pH 12.6), which have half the density and compression strength of the other samples.

The problem with this measurement is the reaction of the metallic Al in the ash with NaOH. This problem appears when the ash is activated with sodium hydroxide solution. The gas release causes the samples to become porous. This paper attempts to show that gas release can be eliminated by the use of additives. This can be clearly seen in the figures in the Table 3, where the M0 sample is clearly expanded and has apparent porosity. The other samples do not exhibit increased porosity. In this case, the other samples do not show gas emission when the alkaline activation additives are used. The porosity of all samples was determined according to the EN 1015-10 determination of dry bulk density of hardened mortar.

Similar values of apparent porosity for bottom ash-based alkali-activated materials were determined by Choeycharoen et al. [23]. The apparent porosity is thus closely related to the compressive strength of the samples. After 28 days of curing, the compressive strength of the samples with lignite coal bottom ash as a precursor and aluminum industry waste NaOH solution as an alkali activator was about 28.91 MPa, and the apparent porosity was about 30.46%.

**Figure 2:**
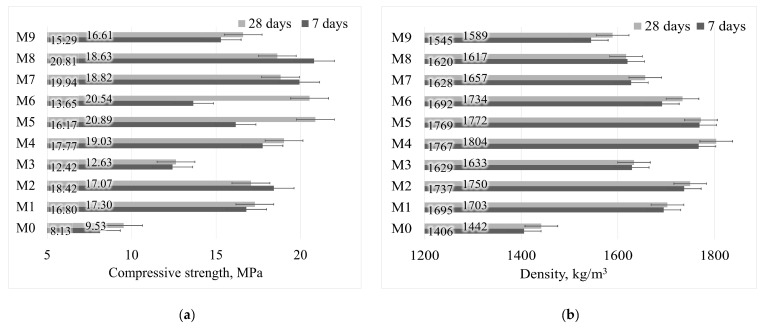
Alkali-activated bottom ash (MSWI) compressive strength (**a**) and density (**b**).

However, the pH of the remaining mixtures did not exceed 12.0, which was controlled by the SW in the activator. Nevertheless, the samples gained mechanical strength and corresponding density (Figure 2a,b).

There were three groups with different water-to-solid ratios: Group 1- M1, M2, M3; Group 2—M4, M5, M6; Group 3—M6, M7, M8. Within each group, the samples differed in the ratio of SiO_2_ to Na_2_O used. The first group showed a higher density result, but no increase in the strength of the samples, which can be explained by the low water ratio in the mixtures and the lack of water. For the same samples such as M2, M7and M8, a reduction in strength after 28 days was determined, not correlated with the density which slightly increased. Wardhono et al. [24] also found a reduction in strength when the density increased. This could be explained by disjoining pressure and self-desiccation effect propagating cracks in alkali-activated ash matrix.

The second group showed the best results in terms of strength and density. In addition, the samples in this group showed the highest changes in strength between 7 and 28 days of hardening. This property was not observed in the other groups. This should have resulted in an optimum mixture composition for maximum results. However, the third group showed the best early results for strength, but the samples had the lowest density. Cracks are visible in the microstructure of these samples, which could account for the lower mechanical strength. Optimally, the use of mixtures with a small amount of SW additive improves the mechanical strength of the samples without causing porosity, but it is necessary to precisely control the water/solid ratio of the mix.

In this study, silica gel waste was used because of its pH level of 2.5. All the samples can be grouped into three groups according to the different amounts of silica gel in the activator solution, namely 0 g, 15 g and 30 g. The pH levels of the activator were 12.6, ~11.75 and 11.50, respectively. The pH of the samples with the highest silica gel residue content was the lowest, which is assumed to result in the lowest solubility of the materials, corresponding to a decrease in the samples’ compression strength.

**Figure 3:**
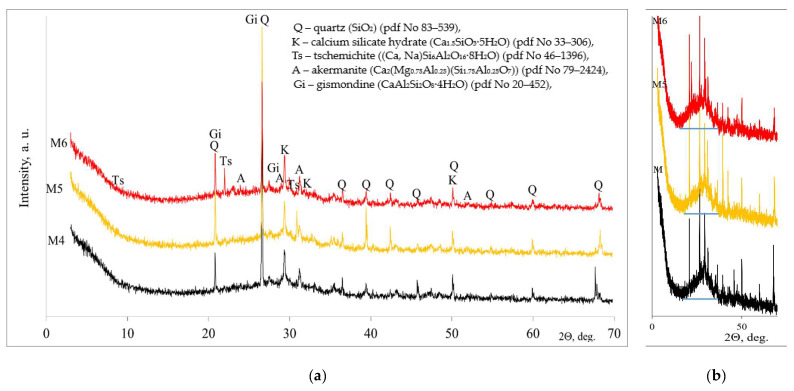
X-ray diffraction patterns of alkali-activated MSWI bottom ash, crystalline composition (**a**) and amorphous halo peak (**b**).

The three samples with the highest strength values were selected and their mineral composition analyzed by XRD analysis (Figure 3a). All the curves show similar peaks, with quartz and calcium silicate hydrate predominating, while akermanite and gismondite were present in negligible amounts. The quartz and akermanite remained unreacted after alkali activation from raw MSWI bottom ash. A study [25] suggests that quartz is almost absent from the geopolymerization reaction and acts as a filter for the matrix. In sample M6, the higher content of aluminosilicate from silica gel waste led to the formation of tschemichite.

After alkaline activation, an amorphous phase was formed in addition to the crystalline compounds. The halo peak of the M4 sample occupied a region between 19.1° and 34.3° (2θ) (Figure 3b). When in the alkali activator a higher amount of soluble glass was added, in the M5 sample, the halo peak occupied a maximum area between 17.8° and 37.9° (2θ). Finally, when the SW content was increased in the triple alkaline activator, the halo peak of the M6 sample occupied a similar area to that of the M4 sample at 16.3°–34.69°. Thus, increasing the amount of soluble glass in the activator system resulted in the formation of a higher amount of N/C-A-S-H gel, which is closely related to the strength of the samples [26].

**Figure 4:**
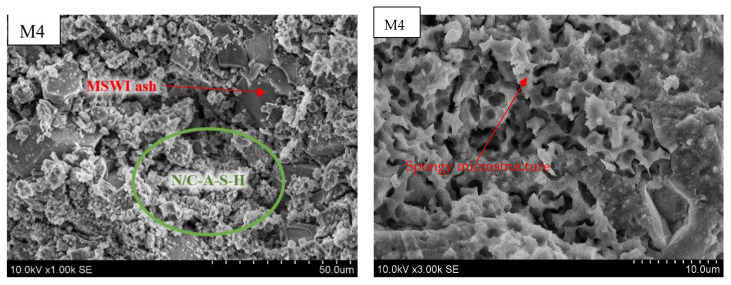

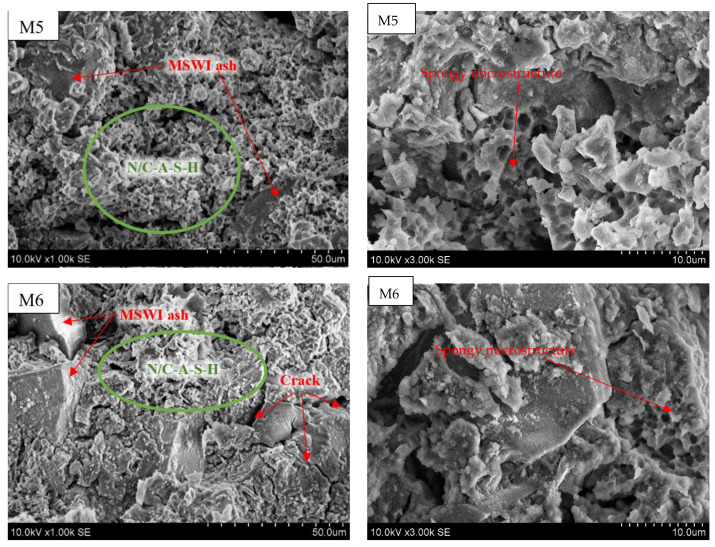
Microstructure of alkali-activated MSWI bottom ash. The samples M4, M5 and M6 is according to Table 2, with different enlargement.

MSWI bottom ash particles, which do not participate in geopolymerization, can be detected in the M4 sample (Figure 4).

These particles are bound by the N/C-A-S-H gel. As the amount of soluble glass in the system increases, a larger amount of gel is formed, which has a positive effect on the strength increase. Finally, the higher silica gel waste content led to the formation of additional crystalline polymerization products, such as tschemichite and gel, which caused the formation of micro-cracks due to drying. This microstructure resulted in slightly lower strength values for sample M6.

The experimental results show that the spongy microstructures encapsulate and bind the unreacted MSWI bottom ash particles, which are the main reaction products for the stabilization of alkali-activated MSWI bottom ash [27].

## 6. Conclusions

The main conclusion of this study was that the lower pH level of the mixtures did not affect the porosity of the samples. At the same time, the results show a doubling of density and mechanical strength. Apparent porosity also decreased from 45.0% to 18.9–26.3%. This allows for the development of an alkali-activated composite, most of which is derived from MSWI bottom ash and relatively inexpensive, with readily available activating agents such as soluble glass, NaOH and silica gel waste.

The mineral composition of all the samples tested was similar, but the amounts varied slightly. Newly formed hydration crystalline products were CSH and gismondite. The higher content of aluminosilicate from silica gel waste led to the formation of tschemichite. Hydration produces amorphous compounds, such as N/C-A-S-H gel, along with crystalline compounds. The largest hump of the N/C-A-S-H gel is closely related to the highest strength. The highest compressive strength of 20.9 MPa after 28 days was reached in the samples with a molar ratio of SiO_2_/Na_2_O = 3.75/1.25.

## Figures and Tables

**Table 1:** Oxide composition of initial material (MSWI ash) according XRF, wt.%.

Oxides	SiO_2_	CaO	Al_2_O_3_	Fe_2_O_3_	MgO	K_2_O	P_2_O_5_	SO_3_	F	Cl	TiO_2_	Na_2_O	Other
**MSWI ash**	42.6	33.62	7.82	3.12	3.37	1.59	1.84	3.17	-	0.35	1.05	0.50	0.97

**Table 2:** Composition of mixtures and quantities of initial materials, in grams.

Mix	MSWI	H_2_O	Soluble Glass	pH Value	NaOH	pH Value	SW	pH Value	W/S	SiO_2_/Na_2_O
**M0**	100	0	40.0	11.4	5.0	12.60	0	12.60	0.20	1.5/1.0
**M1**	100	0	40.0	11.4	5.0	12.60	15	11.75	0.20	3.0/1.0
**M2**	100	0	60.0	11.4	5.0	12.30	15	11.65	0.30	3.75/1.25
**M3**	100	0	60.0	11.4	5.0	12.30	30	11.50	0.30	5.25/1.25
**M4**	100	5	40.0	11.4	5.0	12.62	15	11.75	0.25	3.0/1.0
**M5**	100	5	60.0	11.4	5.0	12.30	15	11.71	0.35	3.75/1.25
**M6**	100	5	60.0	11.4	5.0	12.30	30	11.50	0.35	5.25/1.25
**M7**	100	10	40.0	11.39	5.0	12.63	15	11.75	0.30	3.0/1.0
**M8**	100	10	60.0	11.39	5.0	12.30	15	11.75	0.40	3.75/1.25
**M9**	100	10	60.0	11.39	5.0	12.30	30	11.50	0.40	5.25/1.25

**Table 3:** Apparent porosity of alkali-activated MSWI ash samples.

Mix	M0	M1	M2	M3	M4	M5	M6	M7	M8	M9
	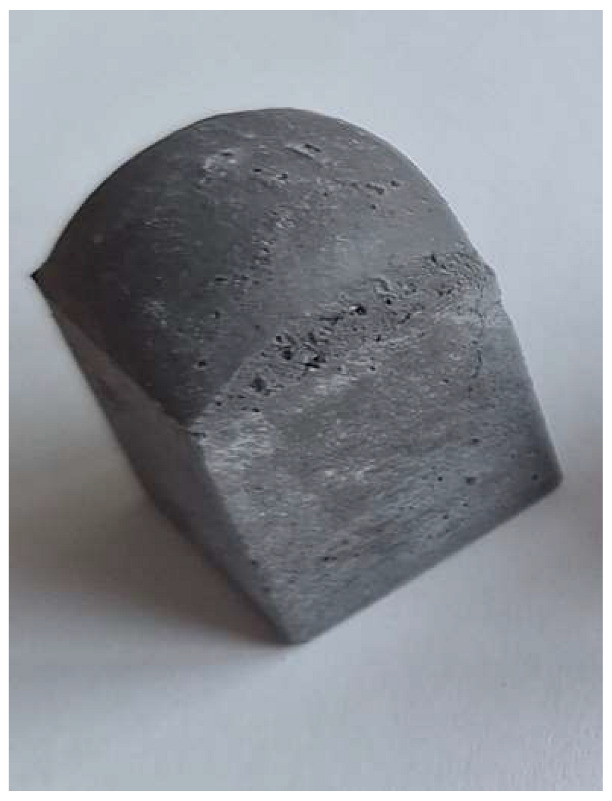	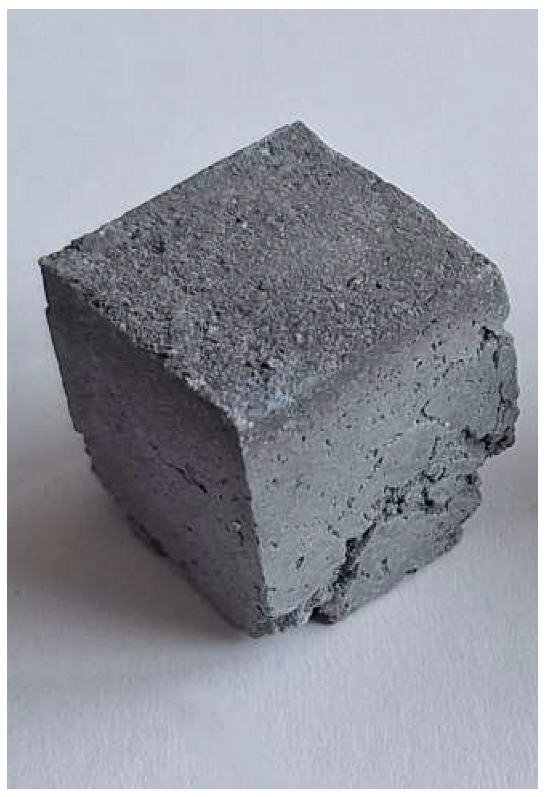	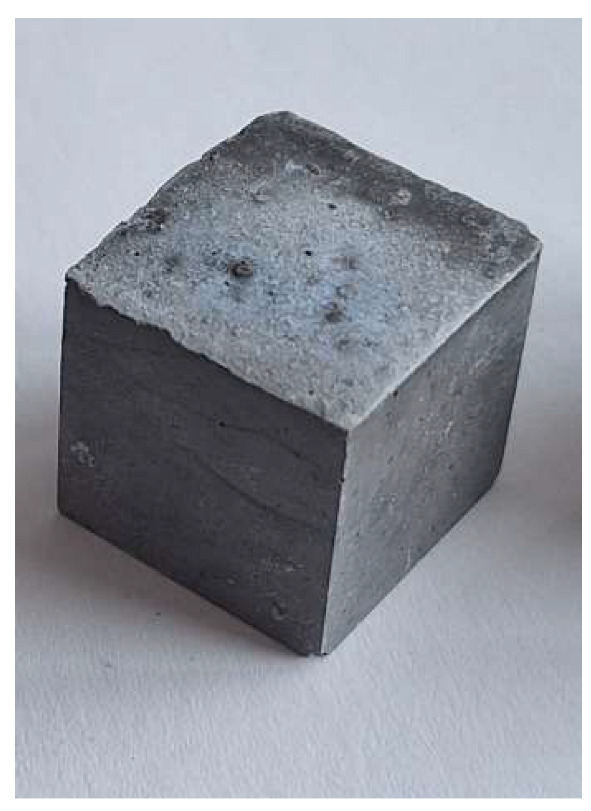	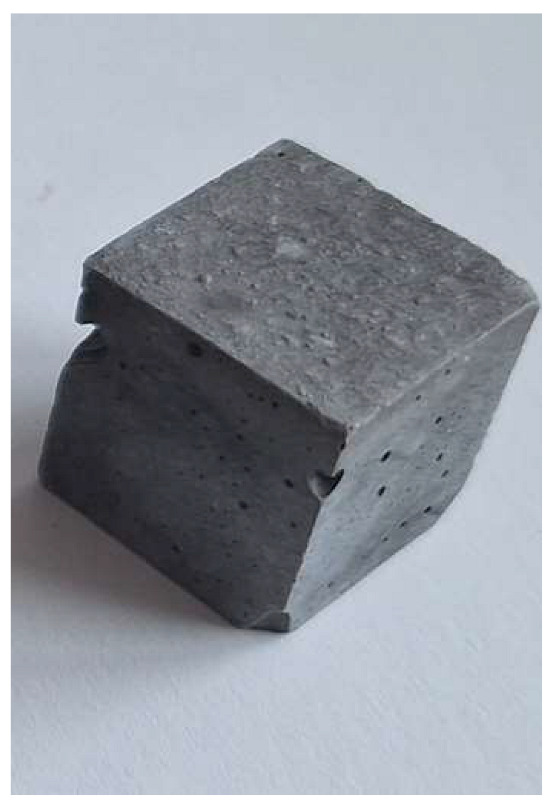	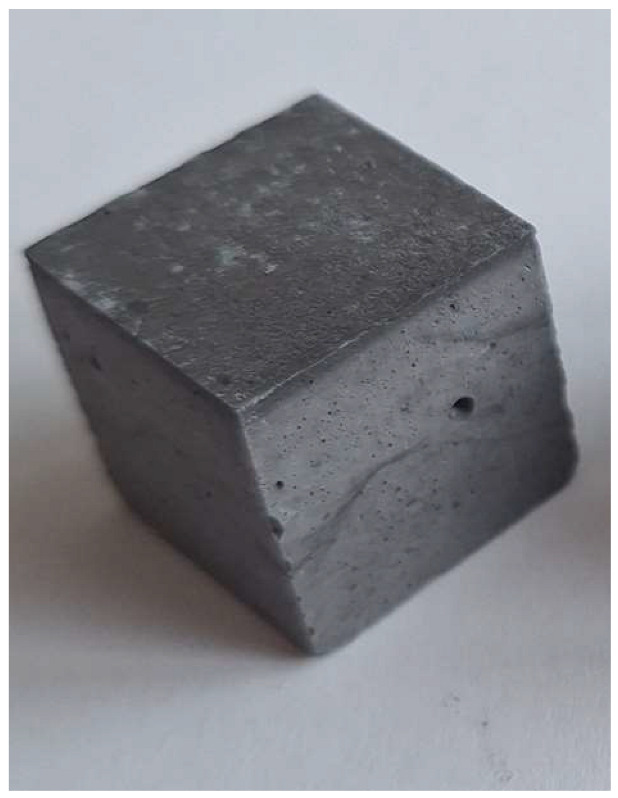	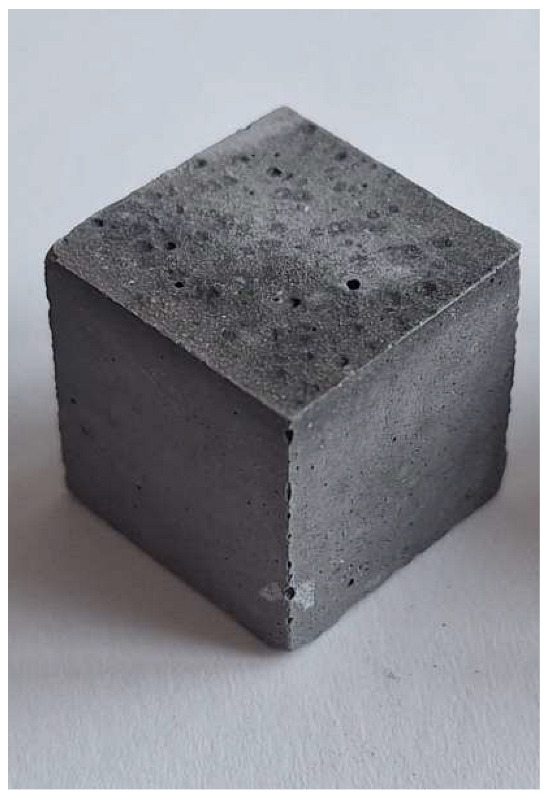	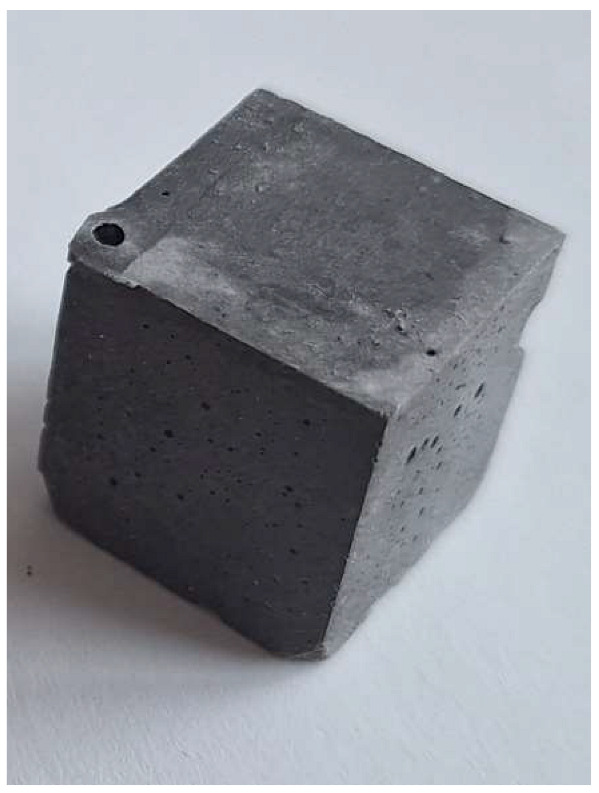	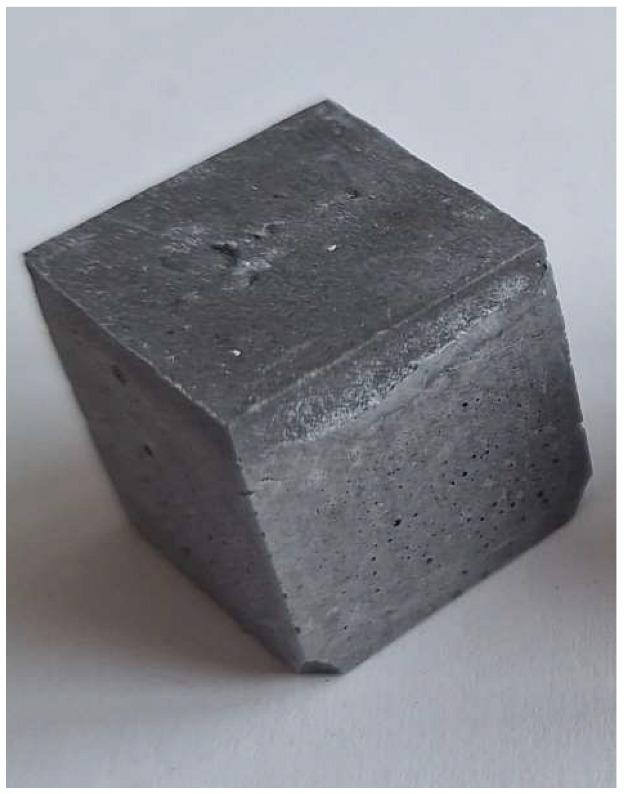	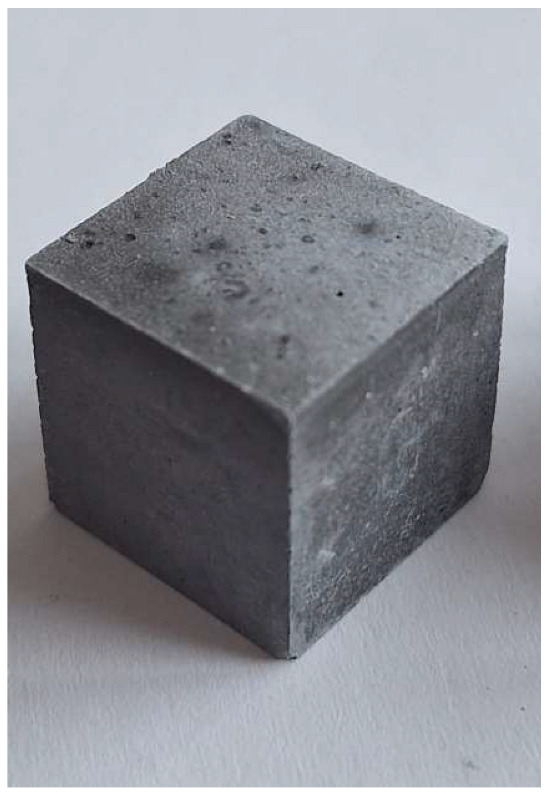	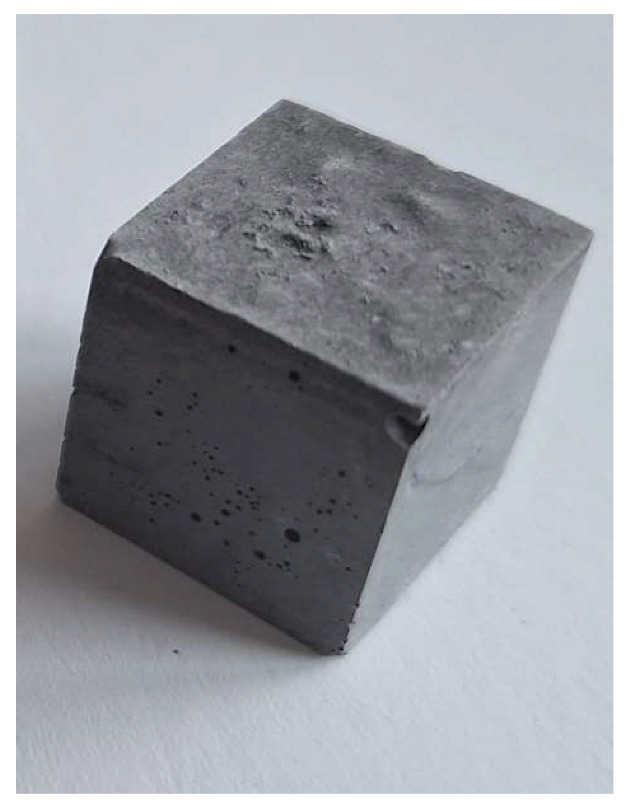
**Porosity, %**	45.0	24.6	24.8	21.7	18.9	20.4	23.4	24.4	25.3	26.3

## Data Availability

The original contributions presented in this study are included in the article. Further inquiries can be directed to the corresponding author.
